# Research on the psychological status of medical staff during the COVID-19 epidemic in China: A longitudinal study

**DOI:** 10.1097/MD.0000000000034750

**Published:** 2023-08-25

**Authors:** Shanshan Li, Shasha Shang, Junrong Wang, Boyi Yang, Wei Jiang

**Affiliations:** a Department of Medicine College, Jiangsu Vocational College of Medicine, Jiangsu Yancheng, China; b Department of cardiovascular, The First Affiliated Hospital of Henan University of CM, Henan Zhengzhou, China; c Department of Obstetrics and Gynecology, Suzhou Ruihua Hospital, Jiangsu Suzhou, China; d Department of geriatrics, Tongji Hospital, Tongji Medical College, Huazhong University of Science and Technology, Hubei Wuhan, China.

**Keywords:** COVID-19, medical staff, psychological status

## Abstract

An online questionnaire, including the Self-Rating Anxiety Scale (SAS) and Self-Rating Depression Scale (SDS), was used to assess the psychological status of medical staff in Wuhan during the COVID-19 epidemic. Lasso-Logistic regression analysis was performed to analyze the risk factors of abnormal psychological status (anxiety or depression). 36.6% of the study subjects experienced anxiety, and 41.5% experienced depression. Female (OR [odds ratio] = 7.22, 95% CI [confidence interval]: 0.58–89.33), basic diseases (OR = 17.95, 95% CI: 1.59–202.49), suspected exposure history (OR = 9.63, 95% CI: 1.40–66.29), smoking (OR = 6.07, 95% CI: 0.38–96.78) were risk factors for anxiety. Female (OR = 5.00, 95% CI: 0.45–55.91), basic diseases (OR = 37.19, 95% CI: 2.70–512.73), suspected exposure history (OR = 5.10, 95% CI: 0.78–33.10), drinking wine (OR = 6.27, 95% CI: 0.38–103.85) were risk factors for depression. The results of the re-sampling evaluation after 2 years showed that some medical staff still showed anxiety (42.4%) and depression (27.3%), and the proportion of females was higher. Early intervention should be carried out, and short-term and long-term intervention plans should be formulated.

## 1. Introduction

Since December 2019, the COVID-19 outbreak in Wuhan has spread rapidly, hurting people lives and health.^[1]^ As of February 11, 2020, 1716 Chinese mainland medical workers were confirmed to have been infected with COVID-19, accounting for 3.8% of the confirmed cases.^[2]^ Le et al (2021) reported a higher risk of exposure among doctors compared to nurses. Moreover, employees in emergency or intensive care units faced a greater risk of exposure than those in non-infected departments.^[3]^

COVID-19 is a significant stress in Wuhan and elsewhere. This is due to overwork, high risk of infection, uncertainty in medical technology and personal capabilities, lack of contact with family members, and isolation during new pneumonia. Serious situations can lead to mental health problems, such as stress, anxiety, depressive symptoms, insomnia, denial, anger, and fear.^[4]^

Studies have shown that in the fight against sudden acute respiratory syndrome (SARS), psychological problems of medical staff gradually emerged: fear and anxiety appeared and eased at the early stage of the epidemic, but depression, psychological symptoms, and post-traumatic stress symptoms appeared later and lasted for a long time.^[5,6]^ A comparative study revealed that nurses in Vietnam exhibit significantly higher levels of depression, stress, intrusion, avoidance, and hyperarousal symptoms compared to their counterparts in Singapore, Malaysia, and Indonesia.^[7]^ Across India, Indonesia, Singapore, Malaysia, and Vietnam, non-medically trained personnel, the presence of physical symptoms, and preexisting medical conditions emerged as significant independent predictors of post-traumatic stress disorder (PTSD), with Vietnam having the highest prevalence of PTSD.^[8]^ Moreover, participants displaying symptoms prior to the study were more likely to be older, have preexisting comorbidities, and test positive for depression, anxiety, stress, and PTSD.^[9]^ A survey involving 906 healthcare workers during the COVID-19 outbreak found a significant association between the prevalence of physical symptoms and psychological outcomes.^[10]^

In a specific study focused on Vietnam, conducted amidst an unprecedented lockdown during the COVID-19 pandemic, minimal impacts were observed on the personal and professional lives of hospital staff. Another survey encompassing 4283 participants from 101 countries revealed that 32.8%, 30.8%, 25.9%, and 24.0% of respondents screened positive for depression, anxiety, stress, and PTSD, respectively. Notably, surgeons specializing in procedures involving the head and neck region exhibited heightened levels of psychological distress.^[11]^

The impact on the mental health of medical staff is not only short-term but also long-term, and the value of practical support and training is meaningful based on previous SARS outbreaks.^[12]^

Practical and comprehensive actions should be taken in time to protect the mental health of medical personnel. Although the current epidemic situation in China has been effectively controlled, there are still reports of new confirmed cases imported from abroad. Under the recent normalization of epidemic prevention and control, occasional sporadic cases occur.^[13]^

At the same time, close to autumn and winter, the high incidence of annually recurring respiratory infectious diseases increases the pressure on epidemic prevention, and the mental health problems of medical personnel cannot be ignored.^[14,15]^

Wuhan City was the first to experience the new coronavirus epidemic. The mental health of medical staff in Wuhan deserves special attention. The Chinese government has made various efforts to reduce the pressure on Wuhan medical staff, such as sending more medical staff to reduce work intensity, adopting strict infection control, providing personal protective equipment, and providing practical guidance. In addition, to reduce the psychological harm of COVID-19 among medical staff, mental health workers in Wuhan are also establishing a psychological intervention group to provide a series of psychological services, including psychological brochures, counseling, and counseling psychotherapy.^[16,17]^ Therefore, we explore the short-term and long-term mental health status of medical staff in Wuhan during the epidemic to provide some reference for better attention and intervention in the psychological crisis of this population.

## 2. Methods

### 2.1. Participants and data collection

**Figure 1.**
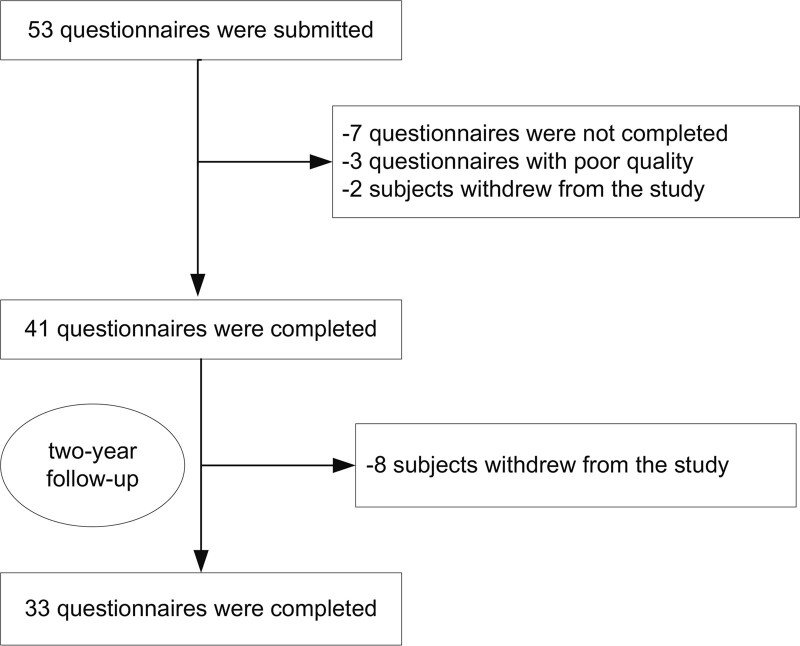
Flow chart of questionnaire screening.

From February 1, 2020 to February 16, 2020, we selected 41 on-the-job medical staff in the hospital general ward as the research participants. A 2-year follow-up of the above subjects was made from January 2, 2022 to January 9, 2022 (Fig. 1). The average age of the subjects was 34.4 ± 5.0 years, including 19 males, accounting for 46.3% of the total number and 22 females, accounting for 53.7% of the total number. See Tables 1 and 2 for the essential data.

**Table 1:** Descriptive statistics for the study variables.

Variable	n	%
Total	41	100
Gender		
Male	19	46.3
Female	22	53.7
Age (yr)	34.4 ± 5.0	/
Marriage		
Married	34	82.9
Unmarried	7	17.1
Education		
College degree or below	3	7.3
Bachelor degree	10	24.4
Master degree or above	28	68.3
Title		
Primary	4	9.8
Intermediate	28	68.3
Senior	9	22.0
Working years		
<5	5	12.2
5–10	27	65.9
>10	9	22
Basic diseases†		
Yes	12	29.3
No	29	70.7
Smoking		
Yes	11	26.8
No	30	73.2
Drinking wine		
Yes	15	36.6
No	26	63.4
Routine exercise		
Often	16	39.0
Occasionally	22	53.7
Never	3	7.3
The only child of the family		
Yes	34	82.9
No	7	17.1
Suspected exposure history‡		
Yes	17	41.5
No	24	58.5
Anxiety state		
Yes	15	36.6
No	26	63.4
Depressive state		
Yes	17	41.5
No	24	58.5

† Basic diseases: Hypertension, neoplastic diseases, hyperuricemia, and one or more of the previous abnormal psychological status (anxiety or depression) episodes, and the condition is stable or clinically cured before the epidemic.

‡ Suspected exposure history: Possible contact with COVID-19 patients.

**Table 2:** General characteristics of research subjects.

Variable	Anxiety state			Depressive state		
	Yes % (n = 15)	No % (n = 26)	*P*	Yes % (n = 17)	No % (n = 24)	*P*
Gender			.06			.07
Male	26.7 (4/15)	57.7 (15/26)		29.4 (5/17)	58.3 (14/24)	
Female	73.3 (11/15)	42.3 (11/26)		70.6 (12/17)	41.7 (10/24)	
Age (M ± SD)	34.5 ± 6.0	34.4 ± 4.5	.96	35.4 ± 5.2	33.8 ± 4.8	.32
Marriage			.71			.93
Married	80.0 (12/15)	84.6 (22/26)		82.4 (14/17)	83.3 (20/24)	
Unmarried	20.0 (3/15)	15.4 (4/26)		17.6 (3/17)	16.7 (4/24)	
Education			.60			.95
College degree or below	6.7 (1/15)	7.7 (2/26)		5.9 (1/17)	8.3 (2/24)	
Bachelor degree	33.3 (5/15)	19.2 (5/26)		23.5 (4/17)	25.0 (6/24)	
Master degree or above	60.0 (9/15)	73.1 (19/26)		70.6 (12/17)	66.7 (16/24)	
Title			.84			.54
Primary	13.3 (2/15)	7.7 (2/26)		5.9 (1/17)	12.5 (3/24)	
Intermediate	66.7 (10/15)	69.2 (18/26)		64.7 (11/17)	70.8 (17/24)	
Senior	20.0 (3/15)	23.1 (6/26)		29.4 (5/17)	16.7 (4/24)	
Working years			.82			.98
< 5 yr	13.3 (2/15)	11.5 (3/26)		11.8 (2/17)	12.5 (3/24)	
5–10 yr	60.0 (9/15)	69.2 (18/26)		64.7 (11/17)	66.7 (16/24)	
> 10 yr	26.7 (4/15)	19.2 (5/26)		23.5 (4/17)	20.8 (5/24)	
Basic diseases†			<.01			<.01
Yes	60.0 (9/15)	11.5 (3/26)		58.8 (10/17)	8.3 (2/24)	
No	40.0 (6/15)	88.5 (23/26)		41.2 (7/17)	91.7 (22/24)	
Smoking			.03			.07
Yes	93.3 (14/15)	61.5 (16/26)		88.2 (15/17)	62.5 (15/24)	
No	6.7 (1/15)	38.5 (10/26)		11.8 (2/17)	37.5 (9/24)	
Drinking wine			.32			.14
Yes	73.3 (11/15)	57.7 (15/26)		76.5 (13/17)	54.2 (13/24)	
No	26.7 (4/15)	42.3 (11/26)		23.5 (4/17)	45.8 (11/24)	
Usual exercise			.51			.94
Often	33.3 (5/15)	42.3 (11/26)		41.2 (7/17)	37.5 (9/24)	
Occasionally	53.3 (8/15)	53.8 (14/26)		52.9 (9/17)	54.2 (13/24)	
Never	13.3 (2/15)	3.8 (1/26)		5.9 (1/17)	8.3 (2/24)	
The only child of the family			.22			.08
Yes	73.3 (11/15)	88.5 (23/26)		70.6 (12/17)	91.7 (22/24)	
No	26.7 (4/15)	11.5 (3/26)		29.4 (5/17)	8.3 (2/24)	
Suspected exposure history‡			<.01			.01
Yes	73.3 (11/15)	23.1 (6/26)		64.7 (11/17)	25.0 (6/24)	
No	26.7 (4/15)	76.9 (20/26)		35.3 (6/17)	75.0 (18/24)	

† Basic diseases: Hypertension, neoplastic diseases, hyperuricemia, and one or more of the previous abnormal psychological status (anxiety or depression) episodes, and the condition is stable or clinically cured before the epidemic.

‡ Suspected exposure history: Possible contact with COVID-19 patients.

The study was approved was obtained from the Ethics Committee of Tongji Hospital, Affiliated with Tongji Medical College of Huazhong University of Science and Technology. Written informed consent was obtained online at the beginning of answering the questionnaire.

### 2.2. Method

The psychological status of the subjects was assessed by an online questionnaire according to self-Rating Anxiety Scale (SAS) and self-Rating Depression Scale (SDS). SAS and SDS scales adopt a 4-level scoring system, with a maximum of 4 points for each question with 20 questions. The higher the score, the greater the degree of anxiety and depression.^[18,19]^ A SAS score higher than 50 is an anxiety state, SDS score higher than 53 is a depression state. Basic diseases: hypertension, neoplastic diseases, hyperuricemia, and one or more of the previous abnormal psychological status (anxiety or depression) episodes, and the condition is stable or clinically cured before the epidemic. Suspected exposure history: possible contact with COVID-19 patients.

Methods of controlling bias included the development of research data collection methods and rigorous quality control methods and using professional questioning techniques by trained personnel.

### 2.3. Follow-up

We again conducted SAS and SDS questionnaires on the above subjects 2 years later.

### 2.4. Statistical method

Spss19.0, Stata15, and R3.5.2 software systems were used for statistical analysis. The measurement data is represented by x̄ ± s, and the counting data is represented by the rate and composition ratio. We performed a nonparametric Mann–Whitney *U* test for categorical variables. *P* < .05 indicates that the difference is statistically significant.

### 2.5. Establishment of Lasso-Logistic regression model

Taking abnormal psychological status (anxiety or depression) as the outcome variable, 13 independent variables were included in the screening. According to the Bayesian information criterion, appropriate Harmonic parameters λ was selected. Then we got the variables with non-zero return coefficients and incorporated them into the final model.^[20]^

## 3. Results

### 3.1. General data characteristics

Among the subjects, 15 had anxiety, accounting for 36.6%; 17 had depression, accounting for 41.5% (Table 1). Women accounted for 73.3% (*P* = .06) in the anxiety population and 70.6% (*P* = .07) in the depression population, but there was no significant gender difference. There were significant differences in basic diseases, smoking, and suspected exposure history. Still, there were no significant differences in age, marital status, education level, professional title, working years, drinking wine, routine exercise, and whether they were the only child of the family or not (Table 2).

### 3.2. Lasso regression analysis

**Figure 2.**
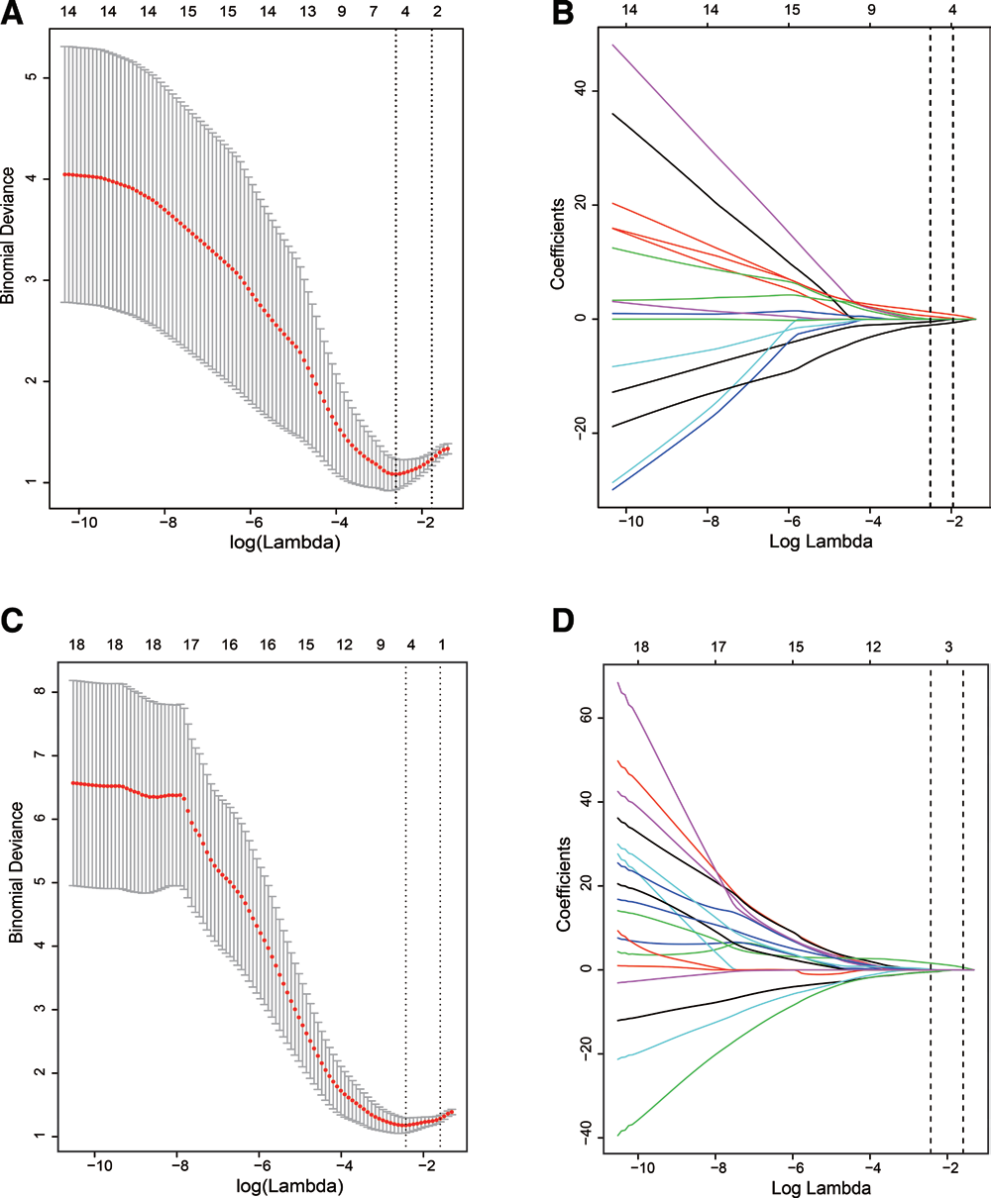
Predictor selection of anxiety (A and B) and depression (C and D) using the minor absolute shrinkage and selection operator (LASSO) regression model. (A) Identification of the optimal penalization coefficient lambda (λ) of anxiety according to Bayesian information criterions. (B) A vertical line was drawn at the optimal λ value, resulting in 4 nonzero coefficients of anxiety. (C) Identification of the optimal penalization coefficient lambda (λ) of depression. (D) Vertical line was drawn at the optimal λ value, resulting in 4 nonzero coefficients of depression.

According to the Bayesian information criterion, taking anxiety state as the outcome variable, we screened the risk factors: gender, basic disease, smoking, and suspected exposure history (Fig. 2A and B). Taking depression as the outcome variable, we screened the risk factors: gender, basic disease, suspected exposure history, and drinking (Fig. 2C and D).

### 3.3. Logistic regression analysis

Taking anxiety status as the outcome variable and gender, basic disease, smoking, and suspected exposure history as the independent variables, we performed logistic regression analysis to obtain the regression equation: y = 1.98*female + 2.89*basic diseases + 2.26* suspected exposure history + 1.80*smoke −11.90 (Table 3). Taking depression as the outcome variable and gender, basic disease, suspected exposure history, and drinking wine as the independent variables, we performed logistic regression analysis to obtain the regression equation: y = 1.61*female + 3.62*basic diseases + 1.63* suspected exposure history + 1.84*drinking wine −11.48 (Table 4).

**Table 3:** Risk factors for anxiety.

Factor	Regression coefficient	OR (95% CI)	*P*
Gender (female)	1.98	7.22 (0.58 89.33)	.12
Basic diseases†	2.89	17.95 (1.59 202.49)	.02
Suspected exposure history‡	2.26	9.63 (1.40 66.29)	.02
Smoke	1.80	6.07 (0.38 96.78)	.20
Constant term	−11.90		.10

CI = confidence interval, OR = odd ratio.

† Basic diseases: Hypertension, neoplastic diseases, hyperuricemia, and one or more of the previous abnormal psychological status (anxiety or depression) episodes, and the condition is stable or clinically cured before the epidemic.

‡ Suspected exposure history: Possible contact with COVID-19 patients.

**Table 4:** Risk factors for depression.

Factor	Regression coefficient	OR (95% CI)	*P*
Gender (female)	1.61	5.00 (0.45 55.91)	.19
Basic diseases†	3.62	37.19 (2.70 512.73)	.01
Suspected exposure history‡	1.63	5.10 (0.78 33.10)	.09
Drinking wine	1.84	6.27 (0.38 103.85)	.20
Constant term	−11.48		.01

CI = confidence interval, OR = odd ratio.

† Basic diseases: Hypertension, neoplastic diseases, hyperuricemia, and one or more of the previous abnormal psychological status (anxiety or depression) episodes, and the condition is stable or clinically cured before the epidemic.

‡ Suspected exposure history: Possible contact with COVID-19 patients.

### 3.4. Follow-up

Two years later, we followed up with the medical staff in the above general ward. There were 33 participants, including 14 males and 19 females. The follow-up results are as follows: 42.4% of the subjects showed anxiety symptoms, of which 64.3% were women; 27. 3% of the subjects showed depression symptoms, of which 66.7% were women.

## 4. Discussion

The outbreak of COVID-19 seriously endangers people life and mental health.^[21]^ In Wuhan, where the epidemic first broke out in China, medical personnel, as the backbone of the fight against the epidemic, withstood multiple psychological shocks, including the epidemic itself and carrying out treatment in high-risk environments. Previous studies reported that the SARS outbreak could have significant psychosocial effects on hospital staff. 29% of the respondents had suffered emotional distress; the rate among nurses was 45%.^[22]^

Our subjects were all from general wards. According to the COVID-19 epidemic prevention and control requirements, the first thing a patient needs to do when admitted to the general ward is to test for COVID-19, whether he is infected or not. However, factors such as being asymptomatic early, having no characteristic imaging changes, or false negative nucleic acid detection in some New Coronavirus infected patients led to potential cross-infection risk in general wards. In addition, the protection level of the general ward was generally lower than that of the fever clinic and isolation ward, which may have placed the medical staff in the general ward more vulnerable to varying degrees of anxiety and depression.

In our study, SAS and SDS scores were used to evaluate the medical staff in some general wards in Wuhan. The results showed that 36.6% of the subjects showed anxiety, and 41.5% showed depression. 80% of the medical staff in anxiety state were co-morbid with depression, while 70.6% of the depressed staff were complicated with anxiety state at the same time. The above results suggested that the epidemic also affected the psychological state of medical staff in general wards, similar to the impact of previous major respiratory infectious diseases on the medical staff.

Our study further analyzed the role of various factors in causing an adverse mental state of medical staff in the general ward and found that gender, basic disease, suspected exposure history, smoking, and drinking were possible risk factors. The incidence rate of female anxiety disorder has been reported to be significantly higher than that of men.^[23]^ Other studies found that after major natural disasters, women had a more significant incidence rate of PTSD than men.^[24]^ However, PTSD was not assessed in the current study.

Among the medical staff involved in this study, 73.3% of anxiety-positive people were female, and 70.6% were in the depression-positive state, indicating that “female” was one of the risk factors leading to anxiety and depression, suggesting that we may need to pay more attention to the psychological state of female medical staff in the epidemic situation.

Previous studies have shown that patients with hypertension had a higher risk of anxiety and depression, and there was a mutually promoting relationship between adverse emotions and hypertension.^[25]^ Depression is recognized as an independent risk factor for hypertension and is more prevalent among individuals with hypertension. Moreover, hypertensive patients exhibit a significantly higher prevalence of negative emotional states compared to non-hypertensive individuals. Patients with other cardiovascular diseases also experience higher levels of depression than the general population. Previous research has highlighted the modifiable association between the severity of depression and triglyceride levels, indicating a positive relationship with the high Framingham Risk Score.^[26,27]^

A prospective study showed that the risk of recurrence of depression in 653 patients with depression after 1 year of cure was 23%, 38% in 2 years, and 47% in 3 years.^[28]^ Vehlings et al investigated 164 patients diagnosed with reproductive testicular tumors but who had reached clinical cure and found that 6% had anxiety and 8% had depression.^[29]^ Domestic studies have also reported that the incidence of depression and anxiety in cancer patients was high (86% of depression and 90% of anxiety). This study showed that abnormal psychological status (anxiety or depression) increased significantly during the epidemic, even if the basic diseases before the epidemic were stable or cured. Further analysis revealed that the history of basic diseases was an independent risk factor for abnormal psychological status (anxiety or depression). Based on the above research results, we believe paying attention to and evaluating medical staff to assess whether there is a psychological overload to provide relevant help and guidance in time is necessary.

Other studies have reported that smoking patients are prone to abnormal psychological status (anxiety or depression), and the incidence of abnormal psychological status increases significantly with tobacco dependence.^[30,31]^ There is also a specific correlation between drinking and depression. The risk of moderate and severe depression in drinkers was higher.^[32]^ In our study, smoking increased the incidence of anxiety, while drinking increased the incidence of depression, and the mechanism might need to be further explored.

The PTSD level of doctors and nurses after their exposure to a novel avian-origin influenza (H7N9) patients was high, up to 20.59%.^[33]^ Another study showed that 39.88% of nurses working in a COVID hospital received a provisional PTSD diagnosis deserving of further analysis.^[34]^ A meta-analysis provides compelling evidence supporting the effectiveness of digital cognitive behavioral therapy for insomnia (dCBT-I) in treating insomnia. Previous studies have shown the efficacy of a 1-week self-guided cognitive behavioral therapy for insomnia (CBTi) in managing acute insomnia and reducing comorbid depressive and anxiety symptoms. Consequently, we urge relevant authorities to consider implementing similar interventions to address the psychological challenges experienced by medical staff during the COVID-19 epidemic.^[35,36]^

These studies showed that the epidemic situation of major respiratory infectious diseases had apparent short-term and long-term psychological effects on the medical staff. Our study also found that 2 years after the outbreak of COVID-19, a considerable proportion of medical staff still had anxiety and depression, varying degrees of impact on work and life.

## 5. Conclusions

This study analyzed the mental health status of medical staff in some general wards of Wuhan hospital and discussed the related risk factors. The study further found that more attention should be paid to female medical staff with a history of underlying diseases and suspicious contacts with confirmed patients. The follow-up after 2 years analyzed the long-term psychological status of high-risk exposed groups. All these provided a reference for establishing a more accurate and practical connection between the psychological assistance the government and hospitals and the medical staff group provided.

The limitation of this study lies in the limited sample size and the bias of the research results. This population psychological evaluation and intervention need more clinical research for further demonstration.

## Acknowledgments

We thank all the participants in this study.

## Author contributions

**Conceptualization:** Shanshan Li, Wei Jiang.

**Data curation:** Shanshan Li, Shasha Shang, Junrong Wang.

**Formal analysis:** Shanshan Li, Shasha Shang, Junrong Wang.

**Investigation:** Shanshan Li, Junrong Wang, Boyi Yang.

**Methodology:** Shanshan Li, Boyi Yang.

**Resources:** Boyi Yang, Wei Jiang.

**Supervision:** Boyi Yang, Wei Jiang.

**Writing – original draft:** Shanshan Li, Shasha Shang.

**Writing – review & editing:** Shanshan Li, Shasha Shang, Boyi Yang, Wei Jiang.
